# Supplementary material for: A UMLS-based spell checker for natural language processing in vaccine safety
Source: BMC Med Inform Decis Mak. 2007 Feb 12;7:3. doi: 10.1186/1472-6947-7-3 (PMC1805499; doi:10.1186/1472-6947-7-3)
Supplement: Additional file 13 — RAP application directory. Sets up directory for RAP (RDF application for PHP) source code [file 1472-6947-7-3-S13.gz › rap/doc/phpdoc/__filesource/fsource_default__vocabularyOWL.php.html]

xml version="1.0" encoding="iso-8859-1"?


File Source for OWL.php


# Source for file OWL.php

Documentation is available at OWL.php

```
1. <?php
2. // ----------------------------------------------------------------------------------
3. // OWL Vocabulary
4. // ----------------------------------------------------------------------------------
5. // Version                   : 0.9
6. // Authors                   : Daniel Westphal (dawe@gmx.de)
7. //
8. // Description               : Wrapper, defining resources for all concepts of the Web
9. //                             Ontology Language (OWL). For details about OWL see:
10. //                             http://www.w3.org/TR/owl-ref/
11. //                                Using the wrapper allows you to define all aspects of
12. //                             the language in one spot, simplifing implementation and
13. //                             maintainence. Working with the vocabulary, you should use
14. //                             these resources as shortcuts in your code.
15. //
16. // ----------------------------------------------------------------------------------
17. // <BR><BR>History:<UL>
18. // <LI>06-25-2004             : $OWL_equivalentClass, $OWL_equivalentProperty, $OWL_Thing, $OWL_Nothing,
19. //                              $OWL_AllDifferent, $OWL_distinctMembers added (auer@informatik.uni-leipzig.de)</LI>
20. // <LI>05-24-2004              : $OWL_DeprecatedClass, $OWL_DeprecatedProperty, $OWL_priorVersion,
21. //                                $OWL_backwardCompatibleWith, $OWL_incompatibleWith (auer@informatik.uni-leipzig.de)</LI>
22. // <LI>03-26-2004              : $OWL:_AnnotationProperty and $OWL_DataRange added (auer@informatik.uni-leipzig.de)</LI>
23. // <LI>02-21-2003             : Initial version</LI>
24. // ----------------------------------------------------------------------------------


27. // OWL concepts

29. $OWL_AnnotationProperty = new Resource(OWL_NS . 'AnnotationProperty');
30. $OWL_AllDifferent = new Resource(OWL_NS . 'AllDifferent');
31. $OWL_allValuesFrom = new Resource(OWL_NS . 'allValuesFrom');
32. $OWL_backwardCompatibleWith = new Resource(OWL_NS . 'backwardCompatibleWith');
33. $OWL_cardinality = new Resource(OWL_NS . 'cardinality');
34. $OWL_Class = new Resource(OWL_NS . 'Class');
35. $OWL_complementOf = new Resource(OWL_NS . 'complementOf');
36. $OWL_Datatype = new Resource(OWL_NS . 'Datatype');
37. $OWL_DatatypeProperty = new Resource(OWL_NS . 'DatatypeProperty');
38. $OWL_DataRange = new Resource(OWL_NS . 'DataRange');
39. $OWL_DatatypeRestriction = new Resource(OWL_NS . 'DatatypeRestriction');
40. $OWL_DeprecatedClass = new Resource(OWL_NS . 'DeprecatedClass');
41. $OWL_DeprecatedProperty = new Resource(OWL_NS . 'DeprecatedProperty');
42. $OWL_distinctMembers = new Resource(OWL_NS . 'distinctMembers');
43. $OWL_differentFrom = new Resource(OWL_NS . 'differentFrom');
44. $OWL_disjointWith = new Resource(OWL_NS . 'disjointWith');
45. $OWL_equivalentClass = new Resource(OWL_NS . 'equivalentClass');
46. $OWL_equivalentProperty = new Resource(OWL_NS . 'equivalentProperty');
47. $OWL_FunctionalProperty = new Resource(OWL_NS . 'FunctionalProperty');
48. $OWL_hasValue = new Resource(OWL_NS . 'hasValue');
49. $OWL_incompatibleWith = new Resource(OWL_NS . 'incompatibleWith');
50. $OWL_imports = new Resource(OWL_NS . 'imports');
51. $OWL_intersectionOf = new Resource(OWL_NS . 'intersectionOf');
52. $OWL_InverseFunctionalProperty = new Resource(OWL_NS . 'InverseFunctionalProperty');
53. $OWL_inverseOf = new Resource(OWL_NS . 'inverseOf');
54. $OWL_maxCardinality = new Resource(OWL_NS . 'maxCardinality');
55. $OWL_minCardinality = new Resource(OWL_NS . 'minCardinality');
56. $OWL_Nothing = new Resource(OWL_NS . 'Nothing');
57. $OWL_ObjectClass = new Resource(OWL_NS . 'ObjectClass');
58. $OWL_ObjectProperty = new Resource(OWL_NS . 'ObjectProperty');
59. $OWL_ObjectRestriction = new Resource(OWL_NS . 'ObjectRestriction');
60. $OWL_oneOf = new Resource(OWL_NS . 'oneOf');
61. $OWL_onProperty = new Resource(OWL_NS . 'onProperty');
62. $OWL_Ontology = new Resource(OWL_NS . 'Ontology');
63. $OWL_priorVersion = new Resource(OWL_NS . 'priorVersion');
64. $OWL_Property = new Resource(OWL_NS . 'Property');
65. $OWL_Restriction = new Resource(OWL_NS . 'Restriction');
66. $OWL_sameAs = new Resource(OWL_NS . 'sameAs');
67. $OWL_sameClassAs = new Resource(OWL_NS . 'sameClassAs');
68. $OWL_sameIndividualAs = new Resource(OWL_NS . 'sameIndividualAs');
69. $OWL_samePropertyAs = new Resource(OWL_NS . 'samePropertyAs');
70. $OWL_someValuesFrom = new Resource(OWL_NS . 'someValuesFrom');
71. $OWL_SymmetricProperty = new Resource(OWL_NS . 'SymmetricProperty');
72. $OWL_Thing = new Resource(OWL_NS . 'Thing');
73. $OWL_TransitiveProperty = new Resource(OWL_NS . 'TransitiveProperty');
74. $OWL_unionOf = new Resource(OWL_NS . 'unionOf');
75. $OWL_versionInfo = new Resource(OWL_NS . 'versionInfo');

77. ?>
```

Documentation generated on Fri, 17 Dec 2004 16:16:40 +0100 by phpDocumentor 1.3.0RC3
